# Optimization of Potassium Supply under Osmotic Stress Mitigates Oxidative Damage in Barley

**DOI:** 10.3390/plants11010055

**Published:** 2021-12-25

**Authors:** Ershad Tavakol, Bálint Jákli, Ismail Cakmak, Klaus Dittert, Mehmet Senbayram

**Affiliations:** 1 K+S Minerals and Agriculture GmbH, Bertha-von-Suttner Str. 7, 34041 Kassel, Germany; 2 Land Surface-Atmosphere Interactions, Technical University of Munich, Hans-Carl-v.-Carlowitz-Platz 2, 85354 Freising, Germany; balint.jakli@tum.de; 3 Faculty of Engineering & Natural Sciences, Sabanci University, 34956 Tuzla, Turkey; cakmak@sabanciuniv.edu; 4 Department of Crop Sciences, Section of Plant Nutrition and Crop Physiology, Georg-August-Universität Göttingen, 37075 Gottingen, Germany; klaus.dittert@agr.uni-goettingen.de; 5 Institute of Plant Nutrition and Soil Science, University of Harran, Osmanbey, 63000 Sanliurfa, Turkey; mehmetsenbayram6@yahoo.co.uk

**Keywords:** K supply, reactive oxygen species (ROS), antioxidant activity, antioxidant enzyme gene expression

## Abstract

Potassium (K) is the most abundant cation in plants, playing an important role in osmoregulation. Little is known about the effect of genotypic variation in the tolerance to osmotic stress under different K treatments in barley. In this study, we measured the interactive effects of osmotic stress and K supply on growth and stress responses of two barley cultivars (*Hordeum vulgare* L.) and monitored reactive oxygen species (ROS) along with enzymatic antioxidant activity and their respective gene expression level. The selected cultivars (cv. Milford and cv. Sahin-91Sahin-91) were exposed to osmotic stress (−0.7 MPa) induced by polyethylene glycol 6000 (PEG) under low (0.04 mM) and adequate (0.8 mM) K levels in the nutrient solution. Leaf samples were collected and analyzed for levels of K, ROS, kinetic activity of antioxidants enzymes and expression levels of respective genes during the stress period. The results showed that optimal K supply under osmotic stress significantly decreases ROS production and adjusts antioxidant activity, leading to the reduction of oxidative stress in the studied plants. The cultivar Milford had a lower ROS level and a better tolerance to stress compared to the cultivar Sahin-91. We conclude that optimized K supply is of great importance in mitigating ROS-related damage induced by osmotic stress, specifically in drought-sensitive barley cultivars.

## 1. Introduction

The world’s climate is currently undergoing an era of rapid change, characterized by increased average temperatures and shifting precipitation patterns. Based on model predictions, there is an increasing risk of extreme drought incidences in many regions (IPCC, 2014: Climate Change-Synthesis Report). Under drought stress conditions, plants have to optimize their transpiration via changing numerous physiological activities in both roots and leaves, including regulation of osmotic potential, hormonal signaling and downregulation of photosynthesis [1,2,3]. The fastest physiological response to osmotic stress is the reduction of stomatal aperture, which decreases the amount of water vapor lost through the stomata [2,4]. This will result in the reduction of stomatal conductance but also decrease internal CO_2_ concentrations in leaf’s mesophyll cells, impairing photosynthesis.

The reduction in photosynthesis as a result of reduced CO_2_ concentration in the chloroplast will favor photorespiration [5]. This process is also combined with the excess production of hydrogen peroxide (H_2_O_2_), which is among one of the most important reactive oxygen species (ROS) in plants. H_2_O_2_ serves as a signaler at low concentrations [6,7], but is a toxic compound at higher levels. H_2_O_2_ also has other generation sources that are unanimously affected under stress conditions, such as drought. For example, H_2_O_2_ can be produced by impaired manganese clusters of photosystem two (PSII) or in both PSII and PSI by the electron donation of iron-sulfur clusters to oxygen [8].

Another ROS species being produced in the photosynthetic cycle is superoxide (O_2_^−^). One of the sources of O_2_^−^ generation is the over-excitation of electrons by the light reactions of photosynthesis, which mainly occurs under excess light stress conditions. This occurs when drought stress is combined with high daylight intensity and is quite common under osmotic stress situations [9]. Under normal conditions, excited electrons will reduce NADP^+^, which is then used by ribulose bisphosphate carboxylase (RuBisCo) to fix CO_2_. However, under stress conditions, the number of excited electrons exceeds normal levels; therefore, the electrons are partially transported to oxygen molecules via ferredoxin in the light reactions producing O_2_^−^ [10]. Similar to H_2_O_2_, O_2_^−^ frequently occurs in the photosynthetic process, even under normal conditions. Nevertheless, when the concentration of O_2_^−^ rises above a natural level, it will become severely destructive [6,11,12].

Initial ROS elevation as a result of biotic or abiotic stresses activates redox signaling in plants leading to an enforced defense mechanism, such as enzymatic antioxidant activity [12,13]. The most important antioxidants in plants are superoxide dismutase (SOD), ascorbate peroxidase (APX), catalase (CAT) and glutathione reductase (GR) [12,14]. Normally, the amount of ROS production is proportional to the amount of detoxification by the antioxidant enzymes [10]. However, under stress situations, such as drought, the balance between ROS production and antioxidant detoxification is disturbed, resulting in excessive ROS production. High rates of ROS will eventuate in cell damage and lead to cell termination [15,16].

When drought stress occurs, the availability of plant nutrients is decisive in plant stress adaptation and stress avoidance. Potassium (K) is the most abundant cation in plants that affects the osmotic potential of plant cells, consequently playing a crucial role in stress mitigation [17,18]. The activation of many enzymes, such as RuBisCO [19], depends on the availability of potassium. A study by Jin et al. [20] has proven the necessity of K in the performance of the photosynthesis apparatus, as it affected the carboxylation rate of RubisCo in hickory seedlings.

Potassium is also important for the transportation of assimilates from source to sink organs [17,21,22], which is mainly performed by changes in the phloem electrical potential [17,23]. Authors reported that the levels of reducing sugars in phloem exudates decreased significantly under continuous K deficiency in beans (*Phaseolus vulgaris* L.) [21]. The deficiency in K will lead to the impairment of RubisCo activity and assimilate translocation causing end-product inhibition of photosynthesis [22]. Drought stress, similar to K deficiency contributes to the disruption of the balance between dark and light reactions of photosynthesis. The latter results in large amounts of excited electrons being transported to oxygen molecules, eventuating in excessive ROS generation [10,14,24,25,26]. Studies reported that supplying the plants with sufficient K nutrition may mitigate the effect of drought stress, mainly by improving assimilate translocation and contributing to osmoregulation [17,22,27,28]. In this regard, low-K plants are found to be highly susceptible to excess light intensity and quickly develop leaf damage symptoms, most probably due to high light-induced ROS formation in chloroplasts [29].

The majority of published work studying the ROS and antioxidant balance of plants under K or osmotic stress was performed as a snapshot shortly after the stress had been initiated [24,30,31,32,33,34,35,36,37]. Very limited studies focused on the time course development of the ROS and antioxidant response [38,39]. As far as we are aware, there were no reports available showing the long-term dynamics of H_2_O_2_, O_2_^−^ and antioxidant activity in the leaf after the start of the osmotic stress period in relation to the varied K supply. Measuring the ROS-antioxidant kinetics will allow us to observe the sensitivity and the severity of plant responses on a daily basis to potassium supply under osmotic stress.

In this paper, we have studied the impacts of adequate and low-K supply on the generation of H_2_O_2_ and O_2_^−^, along with the respective antioxidant response under PEG-induced osmotic stress conditions in two barley cultivars using a hydroponic plant culture. The dynamics of ROS and antioxidant response with the corresponding antioxidants gene expression in our experiment, illustrates how plants balance their antioxidant activity proportional to ROS generation under low potassium supply and osmotic stress.

## 2. Results

### 2.1. Biomass Production

Plants supplied with low-K showed 66% and 53% lower dry matter (DM) in cv. Sahin-91 and cv. Milford in non-PEG treatments, respectively. Inducing osmotic stress by application of PEG to adequate-K treatments also reduced biomass in both cultivars (Table 1). Here, the decrease in biomass in *cv.* Sahin-91 (47%) was more pronounced than in cv. Milford (32%). Expectedly, treating the low-K plants with PEG caused the lowest plant DM yield for both cv. Sahin-91 (83%) and Milford (79%).

As anticipated, adequate K-treated plants had higher K concentrations than the plants with a low-K supply. At a given K treatment, the PEG application did not differently affect plant K concentrations in each cultivar. In the case of low-K supply, the cultivar Sahin-91 contained a slightly higher K concentration than in the cultivar Milford, probably due to less plant biomass and thus the associated “dilution effects”.

The photosynthetic CO_2_^−^ assimilation rate (A_N_) within each cultivar varied between 20 and 30 µmol CO_2_ m^−2^ s^−1^ and was not affected by K supply under non-PEG conditions. Upon application of PEG, A_N_ value dropped significantly, especially under low-K supply in both cultivars. Here, cv. Sahin-91 showed lower assimilation than cv. Milford. Aligned with observations from assimilation rates, the stomatal conductance levels (g_s_) did not show intra-cultivar significance between low-K and adequate-K in cv. Sahin. The latter was, however, significant in cv Milford. Expectedly, the introduction of PEG to the plants reduced the g_s_ rates, especially from adequate-K to low-K in both cultivars. Our earlier report [40] contains a detailed explanation of the assimilation and biomass accumulation of both cultivars.

### 2.2. ROS Concentration, Antioxidant Enzyme Activity and the Respective Gene Expression Levels

#### 2.2.1. Effect of Low-K Supply under Non-Osmotic Stress Conditions

The average concentration of H_2_O_2_ and O_2_^−^ during 11–19 days after onset of the K treatments (11–19 DAO) showed that H_2_O_2_ content increased under deficient K in both cvs. Milford and cv. Sahin-91 (Table 2). The latter, however, in Sahin-91 was considerably higher than Milford. Compared to H_2_O_2_, the fold increase in O_2_^−^ under K deficiency was much higher (over four-fold) in both cultivars. Table 3 shows the *p*-values of Kruskal–Wallis tests for the respective treatments, indicating the significance of K treatments on the generation of H_2_O_2_ and O_2_^−^.

**Figure 1:**
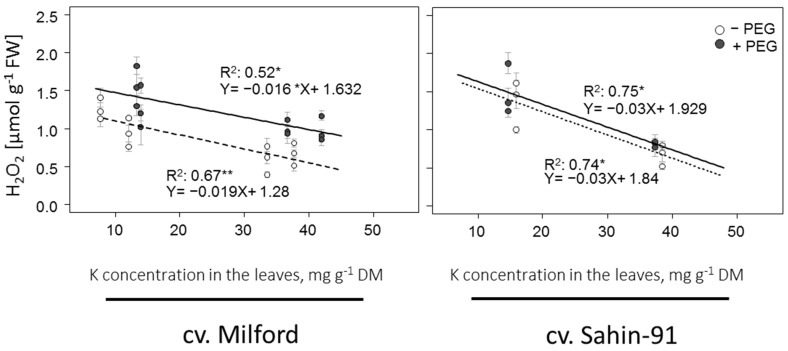
Relationship between H_2_O_2_ and K concentrations in the fully expanded young leaves of two barley cultivars (Sahin-91 and Milford) grown under osmotic stress (filled circles) and no osmotic stress (open circles). Standard errors are depicted on the symbols (*n* = 3). Stars on r squared values show the significance at the level of 0.05 (*) and 0.01 (**).

**Figure 2:**
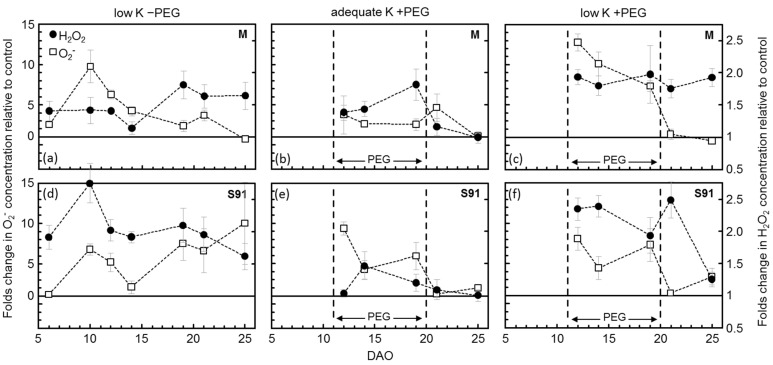
Temporal course of H_2_O_2_ and O_2_^−^ concentration in the youngest fully expanded young leaves of two barley cultivars (Milford, **M**; Sahin-91, **S91**) grown in the nutrient solution under two levels of K supply (adequate K: 0.8 mM K^+^; low K 0.04 mM K^+^) without and with a PEG-induced water deficit (−PEG; +PEG) 5–25 days after the onset of K treatments (DAO). The letters (**a**–**f**) represent each single combination of K-level × PEG-level × barley-variety. On each sampling day, values of the treatments “low K −PEG”, “adequate K +PEG” and “low K +PEG” are expressed as fold changes in comparison to control (“adequate K −PEG” = 100%, represented by the solid horizontal line in each plot). Symbols represent means ± SE (*n* = 6). Significant differences of the measurements are provided in the appendix (Table A1).

Aligned with the ROS response under low and adequate-K treatments, a negative correlation between H_2_O_2_ concentration and K concentration in the fully expanded young leaves was observed (Figure 1). Furthermore, the kinetic ROS concentration during the course of treatment (Figure 2, low-K-PEG) showed high fold changes of ROS content in both cultivars. The dynamics of ROS demonstrate that H_2_O_2_ was constantly higher in Sahin-91 compared to Milford during the whole course of the experiment. The trend in O_2_^−^ is slightly different, being high in Milford at the beginning to middle of the experiment but then rising in Sahin beyond Milford and staying higher in this cultivar from the middle toward the end of the experiment. Overall, on an average basis, the O_2_^−^ concentration was similar in both cultivars during the course of low-K treatment (Table 2).

**Figure 3:**
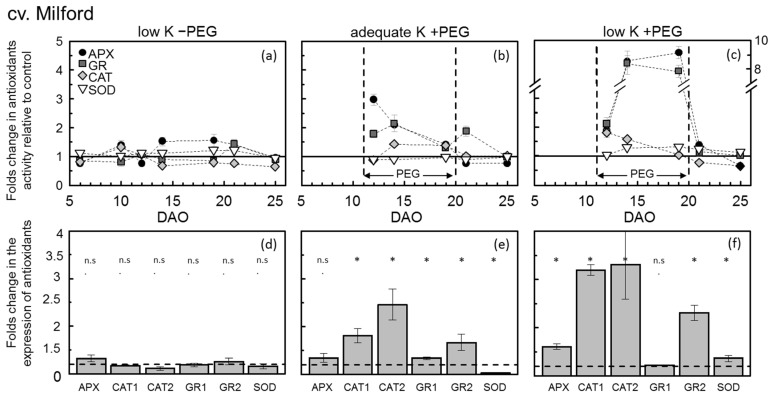
Temporal course of antioxidant (APX, GR, CAT, SOD) activities in fully expanded young leaves (above) 5–25 days after the onset of K treatment (DAO) and the relative gene expression at 14 DAO (below) of barley (cv. Milford) grown in a nutrient solution under two levels of K supply (adequate K: 0.8 mM K+; low K 0.04 mM K+) without and with a PEG-induced water deficit (−PEG; +PEG). On each sampling day, values of the treatments “low K −PEG”, “adequate K +PEG” and “low K +PEG” are expressed as fold changes in comparison to control (“adequate K −PEG” = 10%, represented by the solid horizontal line in each plot). Symbols represent means ± SE (*n* = 6). In the gene expression plots, the dashed horizontal lines similarly represent the control treatment (“adequate K −PEG” = 1), and the bars reflect the fold changes of the selected genes in comparison to control. GR1 and GR2 represent chloroplastic and cytosolic glutathione reductase, respectively. SOD; chloroplastic superoxide dismutase, CAT1 and CAT2; two catalase gene copies and APX; peroxisome type ascorbate peroxidase. Each single combination of K-level × PEG-level × barley-variety for antioxidant activity is shown by the letters (**a**–**c**) and the exact same combination for gene expression is represented by letters (**d**–**f**). The significant expressions are shown with * (*p* < 0.05). All samples were taken at 13:00 h.

**Figure 4:**
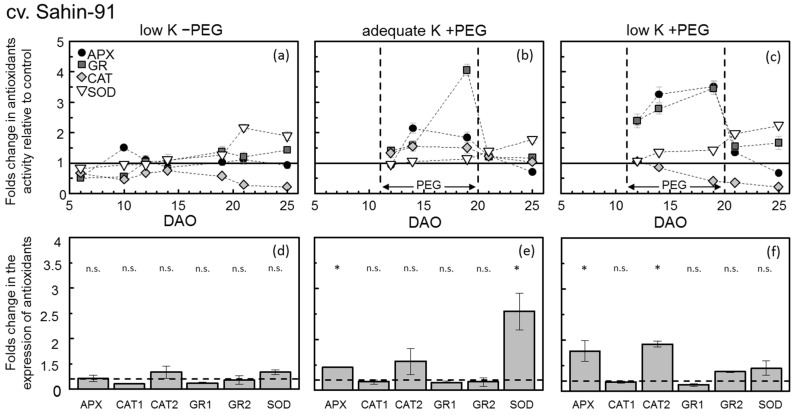
Temporal course of antioxidant (APX, GR, CAT, SOD) activities in fully expanded young leaves (above) 5–25 days after the onset of K treatments (DAO) and the relative gene expression at 14 DAO (below) of barley (cv. Sahin-91) grown in a nutrient solution under two levels of K supply (adequate K: 0.8 mM K+; low K 0.04 mM K+) without and with a PEG-induced water deficit (−PEG; +PEG). On each sampling day, values of the treatments “low K −PEG”, “adequate K +PEG” and “low K +PEG” are expressed as fold changes in comparison to control (“adequate K −PEG” = 100%, represented by the solid horizontal line in each plot). Symbols represent means ± SE (*n* = 6). In the gene expression plots, the dashed horizontal lines similarly represent the control treatment (“adequate K −PEG” = 1), and the bars reflect the fold changes of the selected genes in comparison to control. GR1 and GR2 represent chloroplastic and cytosolic glutathione reductase, respectively. SOD; chloroplastic superoxide dismutase, CAT1 and CAT2; two catalase gene copies and APX; peroxisome type ascorbate peroxidase. Each single combination of K-level × PEG-level × barley-variety for antioxidant activity is shown by the letters (**a**–**c**) and the exact same combination for gene expression is represented by letters (**d**–**f**). The significant expressions are shown with * (*p* < 0.05). All samples were taken at 13:00 h.

The mean APX and GR activity during 11 to 19 DAO (Table 2) was not affected by the K supply (Table 3). The latter was also observed in their Kinect response over the course of the experiment (Figure 3a and Figure 4a). However, CAT activity increased within the first 10 DAO under low-K supply in Milford but significantly decreased afterward (Figure 3).

In Sahin-91, CAT activity levels under low-K supply were constantly below control levels. The SOD activity levels were more or less constant until day 19, which increased significantly afterward compared to the control (Table 2 and Table 3). Similar to the results observed from the activity of antioxidants in low-K treatment, the antioxidant gene expressions in both cultivars showed no changes in expression (Figure 3d and Figure 4d) at the mid-stage of the experiment (14 DAO). The absolute values of ROS and antioxidant enzymes during the whole experimental period are provided in the appendix (Table A1).

#### 2.2.2. Effect of PEG-Induced Water Deficit on Adequate-K Plants

PEG-induced osmotic stress decreased biomass DM and increased leaf H_2_O_2_ and O_2_^−^ concentrations in both cultivars significantly (Table 2). The increase in H_2_O_2_ content in cv. Milford was slightly higher than cv. Sahin-91; however, the O_2_^−^ concentration was three-fold more in cv. Sahin-91 than in cv. Milford. The time-course changes in ROS concentration (Figure 2, adequate K +PEG) showed a several-fold increase in H_2_O_2_ and O_2_^−^ in PEG treatments compared to control shortly after PEG addition, which decreased to normal levels almost instantly after PEG removal in both cultivars.

PEG-induced osmotic stress also increased APX, GR and CAT activity in both barley cultivars; however, the mean increase in APX and CAT activities was highest in cv. Milford (Table 2). The temporal dynamics of antioxidant activity among the cultivars differed significantly during the PEG addition phase. In cv. Milford (Figure 3b), APX activity increased immediately up to three-fold compared to the control treatment and then decreased gradually until the end of the PEG addition phase. On the other hand, GR and CAT activity increased gradually during the PEG period. In cv. Sahin-91, APX and CAT activity increased gradually and then stayed more or less constant, while GR rose constantly until the end of the PEG period (Figure 4b). The activity of all three enzymes in both cultivars decreased rapidly to the control level after PEG removal. In contrast to the other enzyme activities, SOD activity in cv. Milford was significantly lower under PEG-induced osmotic stress and in cv. Sahin-91 it was not affected (Table 3).

The gene expression levels of anti-oxidative enzymes in adequate-K +PEG treatment at 14 DAO indicated that CAT1 and CAT2 expression increased up to 2.5-fold in cv. Milford compared to control treatment (Figure 3e). Both chloroplastic and cytosolic GR genes also showed increased expressions levels; however, the SOD expression level decreased similar to its activity level. In cv. Sahin-91 at 14 DAO (Figure 4e), the expressions of APX and CAT2 genes aligned with their activities were 1.4 and 1.7-fold higher than the control treatment, respectively. In the latter cultivar, SOD was also significantly increased above 2.5-fold of control.

#### 2.2.3. Effect of PEG Introduction to Low-K Treated Plants

Introducing PEG to low-K plants caused an additional decrease in biomass and assimilation rate in both cultivars (Table 1). Expectedly, H_2_O_2_ and O_2_^−^ concentrations were also increased when PEG was added to low-K plants (Table 2, Figure 1). The H_2_O_2_ levels increased up to 2-fold in Milford and almost 2.5-fold in Sahin-91 (Figure 2, low K +PEG). The PEG-induced increase in O_2_^−^ was higher in low-K treated Sahin-91, reaching over 10-fold of control, whereas in Milford, it was 6-fold higher compared to control (adequate-K −PEG). Overall, the highest ROS levels were measured in low-K +PEG-treated cv. Sahin-91. When PEG was removed from the nutrient solution, H_2_O_2_ remained high in both cultivars, whereas O_2_^−^ dropped rapidly to the control levels.

The combination of low-K supply and PEG-induced water deficit significantly increased the activities of antioxidant enzymes in fully expanded young leaves of cv. Milford (Table 2). Here, the amount of increase in APX and GR was drastically higher (reaching 6-fold higher) than in the control rates. SOD and CAT activity also increased in cv. Milford. In cv. Sahin-91, APX and GR activity showed the highest response reaching 3-fold of control. In the latter cultivar, SOD also showed a positive response while CAT was very similar to the “low K −PEG” treatment and was reduced significantly below the control level. The kinetics of the enzyme activities (Figure 3c and Figure 4c) also shows that APX and GR activities drastically increased in the course of the PEG stress period in both cultivars. After removing PEG from the pots (20 DAO), the activity of both APX and GR rapidly reduced to the background levels. SOD activity decreased to its control level in cv. Milford; however, it increased in cv. Sahin-91 over 2-fold of control plants after PEG removal.

The expression of all antioxidant genes at 14 DAO rose significantly in cv. Milford (Figure 3f), corresponding to their observed activity. However, CAT1 and CAT2 seemed to show more expression than the CAT’s enzyme activity. In cv. Sahin-91, aligned with their activity, APX, GR2 and SOD displayed higher expression than control, though only APX’s expression was significant. Moreover, in Sahin-91, CAT2 expression was positive, which was contradictory with its low activity at 14 DAO.

## 3. Discussion

### 3.1. Dry Matter Production Responses to Potassium Supply and PEG Stress

It has been well studied that adequate-K supply enhances crop production and resistance to numerous abiotic and biotic stresses [1,17,22,27,41]. The present study shows a significant decrease in biomass production (53% to 66% in cv. Milford and cv. Sahin, respectively) when K supply switches from optimum to low. Furthermore, the highest yield loss was observed when low-K treated plants were subjected to PEG stress.

One of the known responses to osmotic stress is the increase in abscisic acid (ABA) content in the plants contributing to stomatal closure [42]. The closure of the stoma is performed to avoid unwanted water loss [40,43]. Potassium supply under osmotic stress not only improves plant water uptake but also decreases the ABA content avoiding limitations in stomatal conductance and assimilation. Subsequently, plants with optimal-K supply have better water-use efficiency and assimilation [40,44,45], contributing to higher dry matter production under osmotic stress. Our results showed that cv. Milford had more tolerance to PEG-induced osmotic stress than cv. Sahin-91, especially under adequate-K supply. In agreement with our findings, Feng et al. [46] reported that barley cultivars, which had less K uptake, experienced a yield penalty up to 68% under PEG-induced osmotic stress. Another study on barley [47] reported almost 20% DM loss when K supply was derived from the nutrient solution for 14 days. Studies on other plants, such as rapeseed [26] and wheat [48], showed a significant decrease in biomass between 13% and 30% under low-K supply, both in the absence and presence of PEG.

It is generally proposed that higher biomass yield under sufficient K supply is attributed to improved net photosynthesis [17,20,49,50], higher leaf area production [44,51] and enhanced assimilate translocation [21,52] in crop plants. Furthermore, the reduced capacity of plants to mitigate oxidative stress caused by ROS has also been often discussed as an additional critical factor involved in impairments to the productivity of plants under low-K supply [17,22].

### 3.2. Responses of Reactive Oxygen Species and Antioxidant Activity to K and PEG Treatments

#### 3.2.1. Effects of Low-Potassium Supply under Non-Osmotic Stress Conditions

Increased H_2_O_2_ concentration and O_2_^−^ generation rates under low-K supply have been reported in cotton [53,54], tomato [55], rice [19], corn and Arabidopsis [24] and quinoa [36]. In agreement with these studies, we clearly show that H_2_O_2_ concentration, as well as O_2_^−^ generation rates, increased almost linearly with a decrease in tissue K level (Figure 1). Low-K supply not only leads to limitations in assimilating translocation and mesophyll conductance [21,56], but also negatively affects RuBisCO activity [19]. Such occurrences under low-K supply will eventuate in biochemical limitation to the Calvin cycle and result in excess excited electrons, which form ROS in plants [19,57].

Despite the increased ROS content under K deficiency, only SOD activity on the average of the two cultivars increased significantly, being higher in cv. Milford. CAT activity, on the other hand, reduced significantly. The selected antioxidant gene expressions rates also showed minor changes compared to the control treatment. Although some studies (in agreement with our findings) show a reduction in the activity of antioxidants under low-K supply, so far, the published data has a discrepancy with regards to antioxidants responses. For example, Ahanger and Agarwal [58] showed a reduction in CAT, SOD, APX and GR activity under low-K concentration in wheat plants. Hafsi et al. [34] reported an increase in SOD and APX up to 1.2 and 2.5-fold, respectively, after 26 days in K-deprived Hordeum plants. Wei et al. [48] showed a decrease in CAT and also in SOD activity in low-K supplied wheat plants.

CAT is an enzyme specific to peroxisomes [59]. High rates of photorespiration cause high rates of H_2_O_2_ production in peroxisomes, which are then detoxified by CAT activity. Studies on *Brassica napus*, cotton and rice [19,53,60] demonstrated that photorespiration is reduced by K deficiency. Therefore, we hypothesize that the reduced CAT activity in our study is attributed to the decrease in photorespiration under a low-K supply. Since photorespiration was not measured in our study, a further investigation is required to prove whether, under our experimental conditions, photorespiration has been negatively affected, causing lower CAT activity in the studied plants.

Our data shows that the ROS scavenging mechanisms under low-K supply were not sufficient to mitigate the potassium stress and avoid the chlorosis contributing to significant biomass yield loss in the studied barley cultivars. Furthermore, under low-K supply, cv. Milford had less H_2_O_2_ generation and simultaneously more SOD activity, which explains its higher biomass production compared to cv. Sahin-91.

#### 3.2.2. Osmotic Stress Conditions Induced by PEG to Adequate-K Plants

A drastic increase in leaf ROS generation is reported repeatedly with regards to H_2_O_2_ concentration [28,30,61,62,63] and O_2_^−^ generation rate [62,64,65] under water deficit conditions. A considerable portion of ROS generation is the result of reduced CO_2_ content in the mesophyll cells due to closed stomata or minimized stomatal conductance. The reduction in CO_2_ concentration will cause substrate limitation to the dark reactions of the photosynthesis (Calvin cycle) and initiate excessive O_2_^−^ generation. H_2_O_2_ production under osmotic stress is also enhanced through a variety of sources in the plants, which in some cases act as a signaler against stress [66,67]. For example, H_2_O_2_ production could also be triggered by abscisic acid (ABA) acting as a stomatal closure stimulant [68,69] under water-limited conditions [70]. In our study, although ROS increased under PEG stress, the inter-cultivar changes in H_2_O_2_ were marginal. Nevertheless, the O_2_^−^ generation was significantly higher in cv. Sahin-91, showing that this cultivar is prone to PEG stress to a higher extent compared to cv. Milford.

Excessive ROS generation in the PEG treatment caused an immediate increase in APX, CAT and GR activity in both Sahin-91 and Milford. However, the SOD activity was either slightly reduced in cv. Milford or not affected in cv. Sahin-91. The activity of the respective antioxidants was well-correlated with their gene expressions levels in this study. In agreement with our findings, Harb et al. [71] showed higher CAT and APX activity under drought stress in barley plants, whereas SOD gene expression and activity were either not changed or significantly reduced. Luna [61] showed higher wheat CAT gene expression and enzyme activity under water shortage conditions. Türkan [38] studied the response of *Phaseolus vulgaris* to PEG stress and reported increased CAT activity but a slight reduction in SOD after 14 days of PEG stress conditions. Increased total antioxidant enzymatic activity, with the exception of SOD, has also been reported in rice [72] and alfalfa [32].

It is difficult to draw a general pattern for activation and inhibition of antioxidant activity under osmotic stress conditions; however, in our study, a very high GR activity was observed; thus, we hypothesized that O_2_^−^ generation was mainly detoxified by high GSH produced from the GR cycle [73]. Our study shows that the antioxidant activity under “adequate K +PEG” treatment decreased the absolute ROS concentration. Therefore, we propose that adequate potassium supply under PEG-induced osmotic stress has aided the plants in maintaining their physiological activity and simultaneously detoxified excess ROS damage, avoiding major yield losses in both cultivars.

A comparison of the studied cultivars clearly shows that cv. Milford developed higher antioxidant activity in order to police the excess ROS concentrations and simultaneously preserved the physiological activity at higher rates, resulting in a comparatively less yield loss under osmotic stress conditions (36% DM loss in cv. Milford compared to 47% DM loss in cv. Sahin-91). The latter is supported by our earlier report showing higher assimilation rates and leaf area development of cv. Milford in comparison to cv. Sahin-91 [40].

#### 3.2.3. PEG-Induced Osmotic Stress to Low-K-Treated Plants

When low-potassium-treated plants were exposed to osmotic stress, drastic ROS production occurred. Both cultivars showed the highest ROS content under the combined stress; however, cv. Sahin was more sensitive to low-K content under PEG stress than cv. Milford. The study by Feng [46] showed that the reduced leaf K concentration in barley, due to silenced K transporters (HvAKT2 and HvHAK1), caused higher H_2_O_2_ content in PEG stressed leaves. Knocking out the same gene in rice (OsHAK1) also reduced the K content, causing higher H_2_O_2_ concentrations under PEG stress conditions [74]. Thus, we assume that the combination of potassium deficiency with drought stress triggers ROS generation. The present study disclosed that optimal-K supply is crucial not only under osmotic but also under non-limiting water supply for optimal ROS detoxification.

The high ROS level in our experiment also enhanced APX, GR and SOD activity in both cultivars. However, cv. Milford showed tremendously higher APX and GR activity and positive CAT activity, which was not seen in cv. Sahin-91. This indicated that cv. Milford has a higher antioxidant capacity than cv. Sahin-91, resulting in less photo-oxidation damage and comparatively higher biomass yield under the combined stress situation. The study by Wei et al. [48], in agreement with our findings, showed higher gene expression of APX, SOD, CAT and glutathione peroxidase, contributing to the higher antioxidant activity in wheat genotypes under K deprivation and PEG-induced osmotic stress. In Arabidopsis, knocking out the Zink finger protein (AtRZFP) caused a reduction of K content in the respective lines, which increased SOD, peroxidase and monodehydroascorbate under osmotic stress conditions. In contrast, the studies by Zhu [26] in rapeseed and Chen [74] in rice showed lower antioxidant activity under combined potassium starvation and drought stress conditions. We hypothesize that the reduced catalase enzyme activity in cv. Sahin-91 is due to photo-inactivation under multiple stress conditions. CAT photo-inactivation has been reported in different plants under stress conditions, leading to cell photodamage and resulting in biomass losses [75,76,77,78].

In our study, the activity of most antioxidants or the expression level of related genes were triggered by osmotic stress. Here, the plants provided with sufficient K compared to the K-deprived plants showed lower ROS levels, and therefore, less antioxidant activity. This indicates that K supply under osmotic stress conditions favors mechanisms that mainly avoid and minimize ROS generation (e.g., enhanced photosynthesis activity and better osmoregulation) while simultaneously allowing steady ROS levels to persist in maintaining redox-signaling stress pathways [12].

## 4. Materials and Methods

### 4.1. Experimental Setup

Two barley (*Hordeum vulgare* L.) cultivars, cv. Milford (German origin) and cv. Sahin-91 (Turkish origin), were used for this experiment. After germination, seedlings were grown in hydroponic plant culture for a period of 50 days. The plants were grown in a nutrient solution for a period of 48 days. The average temperature throughout the experimental period was 23 °C at an average relative humidity of 38% and a photosynthetic photon flux density (PPFD) of 400 µmol m^−2^ s^−1^ at plant level, supplied by high-pressure sodium vapor lamps (MASTER Agro 400 W, Philips, The Netherlands). After germination, the seedlings were transferred to an aerated nutrient solution with the following composition: 3 mM NH_4_NO_3_, 1 mM K_2_SO_4_, 1 mM CaCl_2_, 1 mM MgSO_4_, 0.25 mM Ca(H_2_PO_4_)_2_, 0.1 mM Fe-EDTA, 2.5 µM H_3_BO_3_, 2 µM ZnSO_4_, 2 µM MnSO_4_, 0.5 µM CuSO_4_, 0.075 µM (NH_4_)_6_Mo_7_O_24_. Plants were grown in 5-L PVC pots, which had been sealed to exclude water loss due to evaporation, each of them holding two plants. The nutrient solution was renewed in regular intervals of three to four days, depending on plant water consumption. Treatments with two different levels of K supply (0.04 and 0.8 mM K^+^; i.e., low-K and adequate-K) were started 22 days after the plants had been transferred to the solution (DAO = 0, days after onset of treatments). Eleven days after the onset of treatment (DAO = 11), a water deficit was induced by adding 180 g L^−1^ of polyethylene glycol 6000 (PEG) to half of the experimental pots, which instantaneously reduced the osmotic potential of the nutrient solution. PEG was applied in the evening one hour after the photoperiod (20:00). Another 60 g PEG L^−1^ was added to the nutrient solution the next day, one hour before the photoperiod (07:00). Total PEG addition was thus 240 g L^−1^. The remaining pots served as a non-PEG control. Nine days after PEG was introduced to the plants (DAO = 20), PEG was removed from the nutrient solutions, and all experimental pots were again treated equally with no addition of PEG. Each of the 4 treatments was replicated 3 times, resulting in a total of 24 pots for both of the cultivars containing 48 plants (2 plants per pot).

### 4.2. Biomass Harvest and Dry Matter K^+^ Concentrations

At the end of the experimental period (26 DAO), the plants were harvested. The total biomass dry matter (DM), was determined after oven drying of the samples at 65 °C for 48 h. For determination of leaf K concentrations, 100 mg of dried leaf plant material was digested in 4 mL of concentrated HNO_3_ and 2 mL of 30% H_2_O_2_ at 200 °C and 15 bar for 75 min. K concentrations in samples were then measured using an ICP-OES (Vista-RL Simultaneous ICP-OES, Varian Inc., Palo Alto, CA, USA).

### 4.3. Leaf Gas Exchange Measurements

Leaf gas exchange was measured on the youngest fully expanded leaves using a gas exchange system (GFS-3000, Heinz Walz GmbH, Effeltrich, Germany). The cuvette temperature was set to 22 °C, and the relative humidity was 55% at the inlet of the cuvette. The CO_2_ concentration was kept at 380 ppm. An LED array provided a PPFD of 1000 µmol m^−2^ s^−1^. Net assimilation (A) and leaf transpiration were measured and averaged over 5 min after values had stabilized. Stomatal conductance to water vapor (g_s_) was calculated following the model of Caemmerer [79]. Measurements were performed between 9am and 4pm every 3 days during 11–19 DAO, with three replications per treatment.

### 4.4. Hydrogen Peroxide and Superoxide Concentrations

H_2_O_2_ concentrations of the fully expanded young leaves were determined as described by Wolff (1994) and modified by Cheeseman (2009). From 6 to 25 DAO, every 2–3 days 4 leaf discs of 0.78 cm^2^ per plant were taken with a cork borer from selected fully expanded leaves, transferred to 1 mL of acetone acidified with 25 mM H_2_SO_4_ and frozen in liquid nitrogen. At the measurement time, 50 µL of thawed samples were added to 1 mL of FOX solution, which contained 250 µM ferrous ammonium sulfate, 100 mM sorbitol, 100 µM xylenol orange and 25 mM H_2_SO_4_. After incubating the assay at room temperature for 30–45 min. H_2_O_2_ was determined by the difference in absorption at 550 and 850 nm (EPOCH UV-VIS Spectroscopy System, Agilent, Santa Clara, CA, USA).

The superoxide (O2^−^) production rate was measured by the reduction of mono-tetrazolium dye XTT (2,3-bis(2methoxy-4-nitro-5-sulphophenyl)-5-[(phenylamino)carbonyl]-2H-tetrazolium hydrozide, sodium salt) to soluble formazan with superoxide radical anion [80]. The 0.3 mL reaction assay contained 50 mM Tris-HCL buffer, 0.5 mM XTT and a variable sample volume. The production rate of formazan was measured for 5 min at 470 nm, and O_2_^–^ was calculated using the extinction coefficient of 2.16 × 10^4^ M^−1^ cm^−1^.

### 4.5. Anti-Oxidative Enzyme Activities

For measurement of the anti-oxidative enzyme activities involved in ROS scavenging, fully expanded young leaf samples were harvested and immediately frozen in liquid nitrogen. About 0.5 g of samples were homogenized in 5 mL phosphate buffer (pH 7.6), including 1% polyvinylpyrrolidone (PVP) and 0.1 mM EDTA and centrifuged for 20 min at 16,000× *g* at 4 °C. The supernatant was collected and used as a crude extract in the reaction mixtures of the enzyme activity assays. Catalase (CAT) was measured according to Beers and Sizer (1952). The 0.3 mL enzyme assay contained 50 mM phosphate buffer, 0.1 mM EDTA and 10 µL of the crude extract with different dilution levels. The reaction was initiated with H_2_O_2_, and the reduction of H_2_O_2_ concentration was measured spectrometrically (EPOCH, BioTec, USA/8453 UV-VIS Spectroscopy System, Agilent, Santa Clara, CA, USA) following the decrease in absorbance at 230 nm. For the ascorbate peroxidase (APX) assay, the 0.3 mL reaction mixture contained 0.5 mM ascorbic acid, 50 mM phosphate buffer, 1 mM EDTA, 0.5 mM H2O and 10–15 μL of the supernatant. The reaction was started by adding 10 μL of 15 mM of H_2_O_2_ and APX was assayed spectrometrically following the decrease of absorbance at 290 nm (Nakano and Asada 1981). Glutathione reductase (GR) activity was measured by recording the oxidation of NADPH at 390 nm (Halliwell and Foyer 1976). The 0.3 mL reaction mixture contained 0.2 mM nicotinamide adenine dinucleotide phosphate (NADPH), 1 mM GSSG (glutathione disulfide; oxidized form of glutathione), 50 mM K-P buffer (pH 7.6) with 0.1 mM EDTA and 10–15 μL of crude extract. The background was corrected by observing the non-enzymatic oxidation of NADPH in the absence of GSSG. Superoxide dismutase (SOD) activity was determined according to Giannopolitis and Ries [81] with small modifications. The 0.3 mL reaction mixture contained 50 mM phosphate buffer, 0.1 mM EDTA, 50 mM Na2CO3, 12 mM L-methionine, 75 μM nitroblue tetrazolium (NBT), 2 μM riboflavin and 10–20 μL of the enzyme extract. Riboflavin was added at last, and the samples were placed under fluorescent light (4000 lx) for 10 min. Following that, the inhibition of the photoreduction of NBT by SOD was measured at 560 nm. Blank samples with no crude extract were considered, having the highest reaction rate of superoxide with NBT. One unit of SOD activity is defined as the amount of enzyme required to cause 50% inhibition of the rate of NBT reduction at 560 nm.

### 4.6. Gene Expression

Total RNA was extracted from 100 mg of fresh, fully expanded young leaf samples (taken on 14 DAO) using an innuPrep RNA kit (Analytik Jena, Jena, Germany). After treating the samples with DNase I (Sigma), the extracted RNA was reverse transcribed into cDNA using an iScript cDNA Synthesis Kit (Bio-Rad Laboratories Inc., Hercules, CA, USA). The cDNAs were then used as templates for the qPCR reaction using specifically designed primers provided in Table 4. Real-time PCR reactions were performed using (CFX96, BioRad Laboratories, Hercules, CA, USA) and the assay contained 5 µL of Sso Advanced™ Universal SYB^®^ Green superMix (Bio-Rad, Hercules, CA, USA), 2µL of forward and reverse primers, 1 µL of template cDNA and 1 µL of PCR water in a total volume of 10 µL. The thermal cycle’s set up was as follows: 98 °C for 1 min; 44 cycles of 97 °C for 15 s, 58.5 °C for 20 s, and 72 °C for 35 s. The expression levels of antioxidant enzymes were analyzed by the comparative Ct method.

### 4.7. Statistical Analysis

Statistical analyses were performed on the data obtained between 11 and 19 DAO using R 3.3.0 [82]. Since the normality of residuals was not given, a non-parametric Kruskal–Wallis test was used to identify the significant effects of K supply (K), PEG addition (PEG) and the combination of both (K:PEG), relative to the control. Statistical tests were performed with a significance level of α = 0.05.

## 5. Conclusions

Partially relying on their genetic makeup, plants always balance their antioxidant activity according to their redox signaling state. This balance emerges in high activity in some antioxidants while leaving others with comparatively lower activity. In this study, K nutrition proved to be an important factor affecting the ROS concentration in the plants. However, plants seemed to adapt to low-K conditions, taking limited enzyme activity measures to detoxify the ROS compared to PEG-induced osmotic stress. Sufficient K-supplied plants were able to detoxify the ROS under osmotic stress with relatively higher rates than the low-K plants, allowing them to grow and maintain higher biomass. The plants under both low-K supply and osmotic stress showed the highest antioxidant activity in order to detoxify the massive ROS generated. This could be considered as a survival strategy, though, with minimum biomass yield achievement. We conclude that adequate potassium supply under osmotic stress helps the plants to mainly avoid high ROS generation. The reduced ROS under adequate-K supply could be due to improved osmoregulation and maintenance of physiological activity and metabolism in the osmotically stressed plants. Subsequently, the optimally supplied plants are capable of detoxifying the moderate ROS levels via moderate antioxidant activity. Such measures lead to maintenance of yield under sufficient K supply when osmotic stress occurs.

Furthermore, cv. Milford had a higher antioxidant capacity compared to cv. Sahin-91, aiding it to maintain higher biomass yield under osmotic stress conditions. This indicates that the antioxidant enzyme capacity in response to nutrient supply is most likely an intra-species trait and could be used as a reliable phenotyping tool in breeding programs where drought resistance is an important factor in the selection.

## Figures and Tables

**Table 1:** Effect of varied K supply on total dry matter (g pot^−1^), leaf K concentration (mg g^−1^ DM) and assimilation rate AN (µmol CO_2_ m^−2^ s^−1^) of two barley cultivars (Sahin-91 and Milford) under two K application levels (low K: 0.04 and adequate K: 0.8 mM K) and two osmotic stress conditions (no stress: −PEG and with stress: +PEG). The numeric values are the means of treatments provided with standard error (±). Small letters show significance at the level of *p* < 0.05 (*n* = 3) calculated within each cultivar. For detailed and extensive information on this table please refer to [40].

Cultivar	K Application	PEG	Total Plant DM (g pot^−1^)	K Conc. (mg g^−1^ DM)	A_N_ (µmol CO_2_ m^−2^ s^−1^)	g_s_ (mmol m^−2^ s^−1^)
**Sahin-91**	low K	-	10.1 ± 1.9 c	21.9 ± 0.5 b	24.0 ± 2.5 ab	383.5 ± 53.1 a
		+	4.9 ± 1.1 d	24.8 ± 3.7 b	0.7 ± 0.1 c	8.8 ± 0.3 c
	adequate K	-	29.8 ± 0.8 a	42.4 ± 1.8 a	26.4 ± 2.3 a	437.7 ± 32.0 a
		+	15.8 ± 0.5 b	44.4 ± 1.5 a	17.3 ± 2.42 b	146.3 ± 21.7 b
**Milford**	low K	-	18.1 ± 1.7 b	13.2 ± 1.2 b	28.7 ± 2.0 a	408.6 ± 26.4 b
		+	8.5 ± 1.8 c	17.6 ± 0.1 b	4.3 ± 2.1 c	38.5 ± 19.4 d
	adequate K	-	38.9 ± 5.7 a	40.8 ± 1.3 a	28.1 ± 1.0 a	534.6 ± 60.3 a
		+	26.3 ± 2.6 b	39.4 ± 1.8 a	18.7 ± 1.2 b	178.2 ± 19.1 c

**Table 2:** Antioxidant activities (APX, GR, CAT, SOD) and the concentrations of reactive oxygen species (H_2_O_2_, O_2_^−^) in fully expanded young leaves of two barley cultivars (Milford and Sahin-91) grown in the nutrient solution under low (0.04 mM K+) and adequate (0.8 mM K+) K supply without (−PEG) and with (+PEG) PEG-induced water deficit. Means of samples taken between 11 and 19 DAO ± SE are shown (*n* = 18).

K Supply	PEG	APX	GR	CAT	SOD	H_2_O_2_	O_2_^−^
(mM K+)		(µmol H_2_O_2_ g^−1^ FW min^−1^)	(µmol NADPH g^−1^ FW min^−1^)	(mmol H_2_O_2_ g^−1^ FW min^−1^)	(U g^−1^ FW)	(µmol g^−1^ FW)	(nmol g^−1^ FW)
		**Milford**					
0.04	-	1.94 ± 0.17	0.25 ± 0.01	1.26 ± 0.07	138.9 ± 2.2	0.94 ± 0.06	2.63 ± 0.28
0.8	-	1.59 ± 0.18	0.26 ± 0.02	1.52 ± 0.05	121.6 ± 1.9	0.67 ± 0.04	0.61 ± 0.05
0.04	+	9.64 ± 1.20	1.63 ± 0.21	2.21 ± 0.15	146.9 ± 2.7	1.26 ± 0.10	6.34 ± 0.46
0.8	+	3.57 ± 0.42	0.44 ± 0.02	1.88 ± 0.10	111.0 ± 2.0	1.01 ± 0.06	1.83 ± 0.43
		**Sahin-91**					
0.04	-	1.54 ± 0.12	0.27 ± 0.02	0.78 ± 0.03	109.0 ± 3.7	1.36 ± 0.10	2.56 ± 0.41
0.8	-	1.53 ± 0.13	0.23 ± 0.02	1.17 ± 0.05	98.7 ± 2.8	0.67 ± 0.05	0.58 ± 0.06
0.04	+	4.76 ± 0.31	0.66 ± 0.03	0.90 ± 0.09	125.7 ± 2.8	1.49 ± 0.10	3.79 ± 0.54
0.8	+	2.46 ± 0.22	0.53 ± 0.05	1.71 ± 0.10	103.6 ± 3.5	0.81 ± 0.05	3.86 ± 0.57

**Table 3:** Response of antioxidant activities (APX, GR, CAT, SOD) and ROS concentrations (H_2_O_2_, O_2_^−^) to K, PEG and K:PEG. *p*-values of Kruskal–Wallis tests are shown. “+” for K, PEG and K:PEG indicates the following: (K) a significant increase in the respective trait from adequate (0.8 mM K^+^) to low (0.04 mM K^+^) K supply; (PEG) a significant increase in a trait from −PEG to +PEG under adequate K supply; (K:PEG) a significant increase in a trait from −PEG to +PEG under low-K supply. “-“ indicates a significant decrease in the above-mentioned traits. Statistics were performed on fully expanded young leaves samples taken between 11 and 19 DAO (*n* = 18).

	APX		GR		CAT		SOD		H_2_O_2_		O_2_^−^	
	**Milford**											
K	0.062		0.548		0.009	-	<0.001	+	0.002	+	<0.001	+
PEG	<0.001	+	<0.001	+	0.008	+	<0.001	-	<0.001	+	0.004	+
K:PEG	<0.001	+	<0.001	+	<0.001	+	0.025	+	0.016	+	<0.001	+
	**Sahin-91**											
K	0.987		0.142		<0.001	-	0.023	+	<0.001	+	<0.001	+
PEG	0.002	+	<0.001	+	<0.001	+	0.206		0.023	+	<0.001	+
K:PEG	<0.001	+	<0.001	+	0.428		0.002	+	0.255		0.127	

**Table 4:** PCR primers designed to amplify the antioxidant genes catalase (CAT), ascorbate peroxidase (APX), glutathione reductase (GR) and superoxide reductase (SOD) in the barley plants.

Gene	Sequence	Gi-Number
CAT1	5′-GCGGAAAATGAACAGCTTGC-3′	684945
	5′-CATTCACGGGGAGCATCAAG-3′	
CAT2	5′-CGTGGTTGGAAAGAGGGAGA-3′	684947
	5′-ATGCTTGGCTTCACGTTGAG-3′	
APX ^a^	5′-GATTCGTCAGTTTGTCCCCG-3′	15080681
	5′-TTTCAGAGGGTCACGAGTCC-3′	
GR1 ^b^	5′-TACCGAGGAGCAGGCTATTG-3′	157362216
	5′-TCTTGCTTTGTCAACCCAGC-3′	
GR2 ^c^	5′-TCTTTCCGGGGTGAATTCGA-3′	157362218
	5′-ATATGTGCTTCGTCGTGTGC-3′	
SOD ^d^	5′-GACTGGCCCTAATGCAGTTG-3′	304651503
	5′-TGGCGTCGTTACAGGTATGA-3′	
Control	5′-AAGTACAGTGTCTGGATTGGAGGG-3′	DN182500R533
	5′-TCGCAACTTAGAAGCACTTCCG-3′	

a, peroxisome type APX; b, chloroplastic glutathione reductase; c, cytosolic glutathione reductase; d, chloroplast copper/zinc superoxide dismutase.

## Data Availability

Not applicable.

## Appendix A

**Table A1:** Mean values (± SE) of antioxidant response (APX, GR, CAT and SOD) and ROS generation (H2O2 and O_2_^−^) under two K-supply levels (0.04 and 8 mM K) and two PEG levels (no PEG: -, with PEG: +) in two barley cultivars (Sahin-91 and Milford) 6–25 days after the onset of treatments. The letters (a to d) where present, show intra-cultivar significance of the treatments at the level of (0.05).

**DAO**	**K Supply**	**PEG**	**APX**	**GR**	**CAT**	**SOD**
	**(mM K^+^)**		**(µmol H_2_O_2_ g^−1^ FW min^−1^)**	**(µmol NADPH g^−1^ FW min^−1^)**	**(mmol H_2_O_2_ g^−1^ FW min^−1^)**	**(U g^−1^ FW)**
			**Sahin-91**			
6	0.8	-	1.31 ± 0.19	0.37 ± 0.01	2.01 ± 0.07	128.7 ± 1.3
6	0.04	-	0.72 ± 0.10	0.19 ± 0.01	1.35 ± 0.08	106.8 ± 2.0
10	0.8	-	0.77 ± 0.10	0.34 ± 0.03	1.98 ± 0.07	93.1 ± 3.9
10	0.04	-	1.16 ± 0.06	0.19 ± 00.2	0.91 ± 0.05	88.9 ± 6.8
12	0.8	-	1.53 ± 0.22	0.26 ± 0.02	1.21 ± 0.08	108.0 ± 2.3
12	0.04	-	1.69 ± 0.23	0.27 ± 0.02	0.82 ± 0.06	102.7 ± 6.7
12	0.8	+	1.44 ± 0.09	0.37 ± 0.02	1.61 ± 0.16	103.2 ± 5.2
12	0.04	+	3.64 ± 0.23	0.62 ± 0.06	1.27 ± 0.08	115.6 ± 3.8
14	0.8	-	1.33 ± 0.20	0.24 ± 0.03	1.09 ± 0.08	96.2 ± 6.5
14	0.04	-	1.15 ± 0.08	0.26 ± 0.05	0.82 ± 0.04	107.1 ± 5.9
14	0.8	+	2.86 ± 0.22	0.39 ± 0.04	1.68 ± 0.16	102.5 ± 6.4
14	0.04	+	4.32 ± 0.32	0.68 ± 0.04	0.93 ± 0.09	130.5 ± 4.9
19	0.8	-	1.75 ± 0.23	0.20 ± 0.02	1.22 ± 0.07	91.9 ± 2.4
19	0.04	-	1.81 ± 0.21	0.28 ± 0.03	0.70 ± 0.05	117.0 ± 5.7
19	0.8	+	3.21 ± 0.30	0.81 ± 0.04	1.83 ± 0.20	105.1 ± 7.6
19	0.04	+	6.15 ± 0.36	0.69 ± 0.04	0.49 ± 0.04	131.1 ± 3.4
21	0.8	-	1.53 ± 0.27	0.22 ± 0.02	1.54 ± 0.07	48.5 ± 4.7
21	0.04	-	1.72 ± 0.25	0.26 ± 0.04	0.44 ± 0.08	105.1 ± 5.9
21	0.8	+	1.89 ± 0.12	0.26 ± 0.02	1.88 ± 0.04	67.2 ± 3.2
21	0.04	+	2.06 ± 0.17	0.34 ± 0.02	0.55 ± 0.07	95.3 ± 5.5
25	0.8	-	2.45 ± 0.24	0.16 ± 0.02	1.81 ± 0.13	51.9 ± 5.5
25	0.04	-	2.28 ± 0.25	0.23 ± 0.01	0.41 ± 0.06	98.3 ± 7.8
25	0.8	+	1.73 ± 0.20	0.19 ± 0.02	1.87 ± 0.12	92.0 ± 5.9
25	0.04	+	1.66 ± 0.18	0.26 ± 0.04	0.38 ± 0.01	116.5 ± 1.8
			**Milford**			
6	0.8	-	2.33 ± 0.32	0.38 ± 0.02	2.43 ± 0.08	116.6 ± 3.2
6	0.04	-	1.75 ± 0.22	0.32 ± 0.02	1.94 ± 0.10	129.6 ± 1.9
10	0.8	-	1.27 ± 0.16	0.21 ± 0.02	1.50 ± 0.10	122.8 ± 2.7
10	0.04	-	1.79 ± 0.20	0.17 ± 0.03	1.97 ± 0.12	123.4 ± 2.2
12	0.8	-	1.93 ± 0.43	0.25 ± 0.03	1.52 ± 0.09	128.3 ± 3.6
12	0.04	-	1.46 ± 0.12	0.27 ± 0.02	1.59 ± 0.08	142.5 ± 2.4
12	0.8	+	5.71 ± 0.37	0.45 ± 0.04	1.32 ± 0.07	114.1 ± 2.5
12	0.04	+	3.65 ± 0.47	0.53 ± 0.06	2.74 ± 0.17	131.5 ± 1.8
14	0.8	-	1.17 ± 0.15	0.22 ± 0.02	1.59 ± 0.08	117.8 ± 2.5
14	0.04	-	1.77 ± 0.18	0.20 ± 0.02	1.07 ± 0.06	130.0 ± 3.6
14	0.8	+	2.45 ± 0.08	0.46 ± 0.06	2.26 ± 0.09	105.1 ± 3.3
14	0.04	+	9.96 ± 0.50	1.83 ± 0.18	2.50 ± 0.09	150.9 ± 1.2
19	0.8	-	1.67 ± 0.23	0.32 ± 0.02	1.44 ± 0.12	118.8 ± 1.8
19	0.04	-	2.60 ± 0.34	0.27 ± 0.02	1.13 ± 0.09	144.1 ± 3.0
19	0.8	+	2.36 ± 0.17	0.42 ± 0.04	1.98 ± 0.07	114.2 ± 3.1
19	0.04	+	15.32 ± 0.69	2.51 ± 0.13	1.48 ± 0.10	155.7 ± 1.7
21	0.8	-	1.80 ± 0.04	0.13 ± 0.02	1.78 ± 0.13	85.7 ± 6.4
21	0.04	-	2.54 ± 0.32	0.18 ± 0.02	1.33 ± 0.07	103.6 ± 4.4
21	0.8	+	1.37 ± 0.15	0.24 ± 0.02	1.80 ± 0.10	76.0 ± 3.7
21	0.04	+	2.48 ± 0.24	0.14 ± 0.02	1.35 ± 0.09	105.7 ± 4.8
25	0.8	-	2.63 ± 0.33	0.18 ± 0.02	1.77 ± 0.07	104.7 ± 5.7
25	0.04	-	2.49 ± 0.27	0.17 ± 0.02	1.15 ± 0.07	97.7 ± 6.5
25	0.8	+	2.01 ± 0.23	0.18 ± 0.02	1.81 ± 0.11	102.8 ± 4.1
25	0.04	+	1.69 ± 0.19	0.19 ± 0.02	1.15 ± 0.07	118.5 ± 3.0
**DAO**	**K Supply**	**PEG**	**H_2_O_2_**	**O_2_^−^**		
	**(mM K^+^)**		**(µmol g^−1^ FW)**	**(nmol g^−1^ FW min^−1^)**		
			**Sahin-91**			
6	0.8	-	0.57 ± 0.04 ^b^	0.27 ± 0.07 ^a^		
6	0.04	-	1.10 ± 0.11 ^a^	0.31 ± 0.03 ^a^		
10	0.8	-	0.52 ± 0.06 ^b^	0.42 ± 0.14 ^b^		
10	0.04	-	1.42 ± 0.16 ^a^	2.83 ± 0.29 ^a^		
12	0.8	-	0.80 ± 0.05 ^b^	0.58 ± 0.08 ^c^		
12	0.04	-	1.61 ± 0.13 ^a^	3.03 ± 0.66 ^b^		
12	0.8	+	0.83 ± 0.05 ^b^	5.47 ± 0.48 ^a^		
12	0.04	+	1.87 ± 0.14 ^a^	4.71 ± 0.84 ^a,b^		
14	0.8	-	0.52 ± 0.04 ^d^	0.77 ± 0.05 ^c^		
14	0.04	-	1.00 ± 0.05 ^b^	1.59 ± 0.60 ^a,b^		
14	0.8	+	0.77 ± 0.12 ^c^	3.27 ± 0.88 ^a^		
14	0.04	+	1.24 ± 0.09 ^a^	3.39 ± 1.09 ^a^		
19	0.8	-	0.70 ± 0.12 ^b^	0.39 ± 0.08 ^b^		
19	0.04	-	1.46 ± 0.18 ^a^	2.91 ± 0.81 ^a^		
19	0.8	+	0.84 ± 0.09 ^b^	2.30 ± 0.66 ^a,b^		
19	0.04	+	1.35 ± 0.19 ^a^	2.82 ± 0.78 ^a^		
21	0.8	-	0.52 ± 0.10 ^b^	0.30 ± 0.08 ^a^		
21	0.04	-	1.02 ± 0.14 ^a^	1.99 ± 0.82 ^a^		
21	0.8	+	0.57 ± 0.08 ^b^	0.36 ± 0.09 ^a^		
21	0.04	+	1.30 ± 0.15 ^a^	0.40 ± 0.09 ^a^		
25	0.8	-	0.49 ± 0.09 ^b^	0.35 ± 0.17 ^b^		
25	0.04	-	0.80 ± 0.10 ^a^	3.54 ± 1.80 ^a^		
25	0.8	+	0.50 ± 0.04 ^b^	0.70 ± 0.14 ^b^		
25	0.04	+	0.62 ± 0.06 ^a,b^	1.18 ± 0.36 ^b^		
			**Milford**			
6	0.8	-	0.71 ± 0.07 ^a^	0.31 ± 0.05 ^b^		
6	0.04	-	0.99 ± 0.11 ^a^	0.78 ± 0.09 ^a^		
10	0.8	-	0.61 ± 0.05 ^a^	0.28 ± 0.10 ^b^		
10	0.04	-	0.86 ± 0.12 ^a^	2.72 ± 0.57 ^a^		
12	0.8	-	0.81 ± 0.05 ^c^	0.52 ± 0.06 ^c^		
12	0.04	-	1.14 ± 0.02 ^b^	3.27 ± 0.29 ^b^		
12	0.8	+	1.12 ± 0.10 ^b^	1.95 ± 1.25 ^b,c^		
12	0.04	+	1.57 ± 0.10 ^a^	6.69 ± 0.55 ^a^		
14	0.8	-	0.67 ± 0.04 ^c^	0.68 ± 0.08 ^b^		
14	0.04	-	0.76 ± 0.07 ^b,c^	2.89 ± 0.39 ^b^		
14	0.8	+	0.96 ± 0.08 ^b^	1.78 ± 0.24 ^b^		
14	0.04	+	1.20 ± 0.10 ^a^	6 92 ± 1.01 ^a^		
19	0.8	-	0.52 ± 0.07 ^b^	0.70 ± 0.07 ^b^		
19	0.04	-	0.93 ± 0.11 ^a,b^	1.66 ± 0.47 ^b^		
19	0.8	+	0.93 ± 0.13 ^a,b^	1.76 ± 0.50 ^b^		
19	0.04	+	1.02 ± 0.23 ^a^	5.10 ± 0.46 ^a^		
21	0.8	-	0.55 ± 0.11 ^c^	0.18 ± 0.06 ^a^		
21	0.04	-	0.89 ± 0.10 ^a,b^	0.64 ± 0.12 ^a^		
21	0.8	+	0.63 ± 0.07 ^b,c^	0.81 ± 0.30 ^a^		
21	0.04	+	0.96 ± 0.08 ^a^	0.24 ± 0.12 ^a^		
25	0.8	-	0.37 ± 0.02 ^b^	0.73 ± 0.10 ^a^		
25	0.04	-	0.61 ± 0.08 ^a^	0.51 ± 0.08 ^a^		
25	0.8	+	0.37 ± 0.04 ^b^	0.70 ± 0.15 ^a^		
25	0.04	+	0.72 ± 0.05 ^a^	0.40 ± 0.06 ^a^		
